# The Nuclear Cap-Binding Complex Mediates Meiotic Silencing by Unpaired DNA

**DOI:** 10.1534/g3.116.038679

**Published:** 2017-02-06

**Authors:** Logan M. Decker, Hua Xiao, Erin C. Boone, Michael M. Vierling, Benjamin S. Shanker, Shanika L. Kingston, Shannon F. Boone, Jackson B. Haynes, Patrick K.T. Shiu

**Affiliations:** * Division of Biological Sciences, University of Missouri, Columbia, Missouri 65211; † Department of Biology, Barry University, Miami Shores, Florida 33161

**Keywords:** cap-binding proteins (CBPs), meiosis, meiotic silencing by unpaired DNA (MSUD), *Neurospora crassa*, RNA interference (RNAi)

## Abstract

In the filamentous fungus *Neurospora crassa*, cross walls between individual cells are normally incomplete, making the entire fungal network vulnerable to attack by viruses and selfish DNAs. Accordingly, several genome surveillance mechanisms are maintained to help the fungus combat these repetitive elements. One of these defense mechanisms is called meiotic silencing by unpaired DNA (MSUD), which identifies and silences unpaired genes during meiosis. Utilizing common RNA interference (RNAi) proteins, such as Dicer and Argonaute, MSUD targets mRNAs homologous to the unpaired sequence to achieve silencing. In this study, we have identified an additional silencing component, namely the cap-binding complex (CBC). Made up of cap-binding proteins CBP20 and CBP80, CBC associates with the 5′ cap of mRNA transcripts in eukaryotes. The loss of CBC leads to a deficiency in MSUD activity, suggesting its role in mediating silencing. As confirmed in this study, CBC is predominantly nuclear, although it is known to travel in and out of the nucleus to facilitate RNA transport. As seen in animals but not in plants, CBP20’s robust nuclear import depends on CBP80 in *Neurospora*. CBC interacts with a component (Argonaute) of the perinuclear meiotic silencing complex (MSC), directly linking the two cellular factors.

In eukaryotes, RNA polymerase II transcripts undergo multiple processing events to become mature mRNAs that can be translated into polypeptides. An important step is the addition of a 7-methylguanosine to the 5′ end of nascent transcripts. This 5′ cap structure plays a role in multiple aspects of mRNA metabolism, such as protection from exonucleolytic degradation, 3′ end formation, pre-mRNA splicing, export from the nucleus, and translation (Topisirovic *et al.* 2011). These processes are often mediated by proteins that bind to the 5′ cap, also known as cap-binding proteins (CBPs). Two of these, called CBP20 and CBP80, make up the heterodimeric cap-binding complex (CBC), which binds to the 5′ cap and a spectrum of proteins necessary for various RNA processing events. CBC is known to be a key factor for transcription, mRNA stability, pre-mRNA 3′ end processing, splicing, RNA export, the pioneer round of translation, and nonsense-mediated mRNA decay (NMD) (Gonatopoulos-Pournatzis and Cowling 2014).

Besides the aforementioned functions, CBC is also implicated in small RNA-mediated silencing (Gregory *et al.* 2008; Kim *et al.* 2008; Laubinger *et al.* 2008; Gruber *et al.* 2009; Sabin *et al.* 2009). In *Neurospora crassa*, an RNA interference (RNAi) system known as meiotic silencing by unpaired DNA (MSUD) exists to suppress expression from unpaired genes during sexual development (Shiu *et al.* 2001). A working model for MSUD begins with the detection of an unpaired DNA region during meiotic prophase I (with the help of suppressor of ascus dominance-6 or SAD-6, a presumptive homology search protein; Samarajeewa *et al.* 2014). A single-stranded aberrant RNA (aRNA) is transcribed from the unpaired DNA and subsequently exported to the perinuclear region. There, the aRNA encounters the meiotic silencing complex (MSC) (Decker *et al.* 2015), which contains several RNAi-related proteins. One of these is SAD-1, the RNA-directed RNA polymerase responsible for converting the aRNA into double strands (Shiu and Metzenberg 2002). Working alongside SAD-1, a helicase known as SAD-3 may assist in the RNA synthesis (Hammond *et al.* 2011a). The resulting double-stranded RNA (dsRNA) is then cut into small interfering RNAs (siRNAs) by the DCL-1 Dicer-like protein (Alexander *et al.* 2008). The QIP (QDE-2-interacting protein) exonuclease converts the siRNA duplexes into single strands, which subsequently guide the SMS-2 (suppressor of meiotic silencing-2) Argonaute to target complementary mRNAs for silencing (Lee *et al.* 2003; Xiao *et al.* 2010). SAD-2 is thought to act as a scaffold protein and tether the aforementioned MSC components to the perinuclear region (Shiu *et al.* 2006; Decker *et al.* 2015). Two other proteins, SAD-4 and SAD-5, are essential for the production of siRNAs; however, their precise functions in MSUD remain unknown (Hammond *et al.* 2013a, b). In this study, we have shown that cap-binding proteins CBP20 and CBP80 also play a role in MSUD.

## Materials and Methods

### Fungal manipulation and genotypic information

Standard techniques from the *Neurospora* protocol guide were used throughout this work (http://www.fgsc.net/Neurospora/NeurosporaProtocolGuide.htm). Strain names and genotypes are listed in Table 1. Knockouts and other markers were obtained from the Fungal Genetics Stock Center (FGSC) (McCluskey *et al.* 2010) and the Neurospora Functional Genomics Group (Colot *et al.* 2006). Fungal isolates were grown on Vogel’s medium (Vogel 1956). Crosses were performed on synthetic crossing medium of Westergaard and Mitchell (1947).

**Table 1:** *Neurospora* strains used in this study

Strain	Genotype
F2-01	*fl A* (FGSC 4317)
F2-29	*rid r*^Δ^::*hph*; *fl A*
F6-15	*rid r*^∆^::*hph*; *fl*; *cbp20*^∆^::*hph*; *cbp80*^∆^::*hph A*
F6-19	*fl*; *cbp80*^∆^::*hph a*
F6-20	*rid r*^∆^::*hph*; *fl*; *cbp20*^∆^::*hph A*
F8-02	*fl*; *cbp20*^∆^::*hph A*
F8-04	*fl*; *cbp20*^∆^::*hph*; *cbp80*^∆^::*hph A*
P3-08	Oak Ridge wild-type (WT) *a* (FGSC 2490)
P6-59	*sad-1^∆^*::*hph rid his-3*^+^::*yfpn A*
P8-25	*rid his-3*^+^::*yfpc*; *inv sad-2^RIP^ a*
P15-36	*rid*; *mus-52*^∆^::*bar*; *yfpc-sms-2*::*hph a*
P18-19	*rid his-3*^+^::*yfpc-sad-1*; *yfpn-sms-2*::*hph A*
P18-21	*sad-1*^Δ^::*hph rid his-3*^+^::*yfpc-sad-1*; *yfpn-sms-2*::*hph a*
P20-34	*rid mCherry-nup120*::*hph his-3*; *mus-51*^∆^::*bar*; *gfp-cbp80*::*hph A*
P20-35	*rid mCherry-nup120*::*hph his-3*; *mus-51*^∆^::*bar a*
P20-38	*cbp80*^∆^::*hph a*
P20-39	*cbp80*^∆^::*hph A*
P20-42	*rid r*^∆^::*hph*; *cbp80*^∆^::*hph A*
P20-44	*rid his-3*; *mus-52*^∆^::*bar mCherry-cbp20*::*nat*; *mus-51*^∆^::*bar*; *gfp-cbp80*::*hph a*
P20-45	*rid his-3*; *mus-52*^∆^::*bar mCherry-cbp20*::*nat*; *mus-51*^∆^::*bar*; *gfp-cbp80*::*hph A*
P20-47	*cbp20*^∆^::*hph a*
P20-68	*cbp20*^∆^::*hph*; *cbp80*^∆^::*hph a*
P21-10	*rid*; *mus-52*^∆^::*bar yfpn-cbp20*::*nat*; *yfpc-cbp80*::*hph a*
P21-11	*rid*; *mus-52*^∆^::*bar yfpn-cbp20*::*nat*; *yfpc-cbp80*::*hph A*
P21-52	*rid*; *yfpc-sad-5*::*hph A*
P21-53	*rid*; *yfpn-cbp80*::*hph*; *yfpc-sad-5*::*hph a*
P21-54	*rid his-3*; *mus-52*^∆^::*bar*; *yfpn-cbp80*::*hph*; *yfpc-sad-5*::*hph A*
P23-26	*rid his-3*; *mus-52*^∆^::*bar yfpn-cbp20*::*nat a*
P23-28	*rid gfp-nup120*::*hph his-3*; *mCherry-cbp20*::*nat*; *cbp80*^∆^::*hph a*
P23-29	*mCherry-cbp20*::*nat*; *cbp80*^∆^::*hph A*
P25-55	*rid his-3*; *mus-52*^∆^::*bar yfpn-cbp20*::*nat A*
P25-56	*rid*; *mus-52*^∆^::*bar*; *yfpc-cbp80*; *yfpn-sms-2 A*
P25-57	*rid*; *mus-52*^∆^::*bar*; *yfpc-cbp80*; *yfpn-sms-2 a*
P25-58	*cbp80*^∆^::*hph A* (FGSC 22440)
P25-59	*cbp80*^∆^::*hph a* (FGSC 22441)

Description of genetic loci can be found in the e-Compendium (http://www.bioinformatics.leeds.ac.uk/∼gen6ar/newgenelist/genes/gene_list.htm).

### Library screening and MSUD suppression assay

Screening of the knockout library for MSUD-deficient mutants and the subsequent quantitative analysis of silencing suppression were performed using established protocols (Hammond *et al.* 2011a; Samarajeewa *et al.* 2014).

### RNA expression analysis

For comparison of RNA transcripts, *Neurospora* vegetative (SRR080688, SRR081479, SRR081546, and SRR081586) and sexual (SRR957218) RNA-seq datasets were downloaded from the European Bioinformatics Institute's European Nucleotide Archive (Ellison *et al.* 2011; Samarajeewa *et al.* 2014). All datasets were aligned to predicted *Neurospora* transcripts using Bowtie 2 v2.2.3 (Langmead and Salzberg 2012). RNA levels (in fragments per kilobase of exon per million mapped reads or FPKM) were calculated using eXpress v1.5.1 and Microsoft Excel (Roberts and Pachter 2013).

### Transformation and strain construction

Transformation by electroporation of conidia (asexual spores) was conducted using the technique of Margolin *et al.* (1997). All fluorescently tagged strains described in this study were constructed using the double-joint polymerase chain reaction (DJ-PCR) method (Hammond *et al.* 2011b; Samarajeewa *et al.* 2014).

### Genotype screening and strain confirmation

Genomic DNA was isolated from conidia (Henderson *et al.* 2005) or vegetative hyphae (Qiagen DNeasy Plant Mini Kit). PCR-based confirmation of genotypes was performed with the Promega GoTaq Green Master Mix or the Roche Expand Long Range dNTPack.

### Bimolecular fluorescence complementation (BiFC)

BiFC is an *in vivo* protein–protein interaction assay, and it relies on the reconstitution of the yellow fluorescent protein (YFP) when its nonfluorescing halves are brought together by two interacting proteins (Hu *et al.* 2002; Bardiya *et al.* 2008). Generation of BiFC constructs was as described by Hammond *et al.* (2011b).

### Photography and microscopy

Z-stack pictures of protoperithecia (female structures) were taken using a Leica M205 FA stereomicroscope with a Leica DFC345 FX camera. Representative images of asci (spore sacs) and perithecia (fruiting bodies) were obtained using an Apple iPhone 5 with a Magnifi photoadapter (Arcturus Labs, Palo Alto, CA) on a Vanguard 1231CM microscope. An Olympus BX61 was used for fluorescent microscopy. Preparation and visualization of asci (≥10/cross) were performed as described (Alexander *et al.* 2008; Xiao *et al.* 2010), with similar exposure times used across samples (50–150 ms for DAPI and 500–800 ms for fluorescent proteins).

### Data availability

Strains are available upon request. The authors state that all data necessary for confirming the conclusions presented in the article are represented fully within the article.

## Results

### Identification of two CBPs as MSUD players

To identify novel MSUD components, we have developed a high-throughput screen of the *Neurospora* knockout library for silencing mutants (Hammond *et al.* 2011a). Preliminary data from this screen identified two additional strains [FGSC 22440 (*A*) and 22441 (*a*)] that appear to be MSUD-deficient. These strains correspond to deletion mutants of *ncu04187*, also known as *cbp80*. PCR amplification was used to verify the *cbp80* deletion in these strains before its effect on silencing was evaluated in a quantitative MSUD assay.

**Figure 1:**
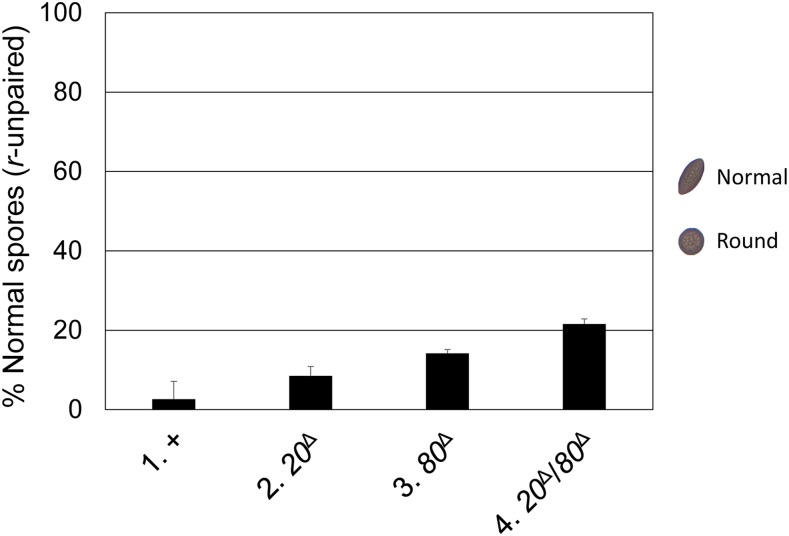
The absence of CBC correlates with a reduction in MSUD activity. Here, crosses heterozygous for *r*^∆^ were examined. When MSUD is proficient, it silences the unpaired *r^+^* gene, which results in the production of mostly round ascospores [with only 2.6% of the progeny being normal (American football-shaped); cross 1]. When both parents lack CBP20 and CBP80, MSUD becomes deficient and considerably more normal ascospores (21.6%) can be observed (cross 4). Crosses homozygous for either single *cbp* knockout exhibit a more modest MSUD suppression (with 8.5% and 14.2% normal ascospores; crosses 2 and 3, respectively). In comparison, MSUD appears to be completely suppressed in a *sad-5*-null background (Hammond *et al.* 2013b). Crosses were performed in triplicate, with an average of ∼100 ascospores/cross examined. +, wild type (WT) at *cbp* loci. 1, F2-29 × P3-08. 2, F6-20 × P20-47. 3, F6-19 × P20-42. 4, F6-15 × P20-68.

A *Neurospora* cross typically produces American football-shaped ascospores (sexual spores). However, if the *Round spore* gene were unpaired (and hence silenced), the cross (*r*^+^ × *r*^∆^) would produce predominantly round ascospores (Shiu *et al.* 2001). This aberrant phenotype can be alleviated by the deletion of an MSUD gene. As seen in Figure 1, the removal of *cbp80* increases the percentage of normal ascospores fivefold. This suggests that efficient silencing of unpaired genes requires CBP80.

Because CBP80 is the large subunit of CBC, it is possible that its known binding partner, CBP20, also plays a role in silencing. Indeed, deletion of *cbp20* (*ncu00210*) also affects MSUD, albeit to a lesser extent (Figure 1). When both CBPs are absent in a cross, the percentage of normal ascospores increases eightfold. These data suggest that CBC, a multifaceted eukaryotic factor, mediates MSUD.

### Expression of CBC genes during both vegetative and sexual cycles

To determine the expression patterns of *cbp20* and *cbp80*, we examined their transcripts via RNA-Seq datasets (see *Materials and Methods*). While the MSUD-exclusive genes examined here have relatively low vegetative expression (as compared to their sexual expression), *cbp20* and *cbp80* have robust expression levels in both lifecycles (Table 2). These data are consistent with the notion that CBC plays a role in both sexual and asexual stages. Indeed, it has been shown that CBC is important for NMD during vegetative growth in *Neurospora* (Zhang and Sachs 2015).

**Table 2:** Expression of RNA silencing genes

Gene Name	Gene No.	Vegetative Expression (FPKM)	Sexual Expression (FPKM)
Cap-binding complex			
*cbp20*	*ncu00210*	105.3384	214.7519
*cbp80*	*ncu04187*	63.1688	59.2896
Housekeeping			
*actin*	*ncu04173*	2638.3425	905.4051
*β-tubulin*	*ncu04054*	1222.0460	223.5137
MSUD			
*sad-1*	*ncu02178*	0.3684	14.4495
*sad-2*	*ncu04294*	0.0000	38.5137
*sad-3*	*ncu09211*	0.0360	18.5717
*sad-4*	*ncu01591*	0.5295	8.5876
*sad-5*	*ncu06147*	0.0000	13.2559
*sms-2*	*ncu09434*	0.0496	673.0190
MSUD/Quelling			
*dcl-1*	*ncu08270*	4.4300	31.0978
*qip*	*ncu00076*	18.6841	107.2514
Quelling			
*dcl-2*	*ncu06766*	4.9037	51.8974
*qde-1*	*ncu07534*	17.1798	23.2177
*qde-2*	*ncu04730*	96.9095	287.2828
*qde-3*	*ncu08598*	8.1519	4.6163

Quelling is the vegetative RNA silencing mechanism that targets tandem transgenes. FPKM, fragments per kilobase of exon per million mapped reads.

### Loss of CBC impairs sexual development

**Figure 2:**
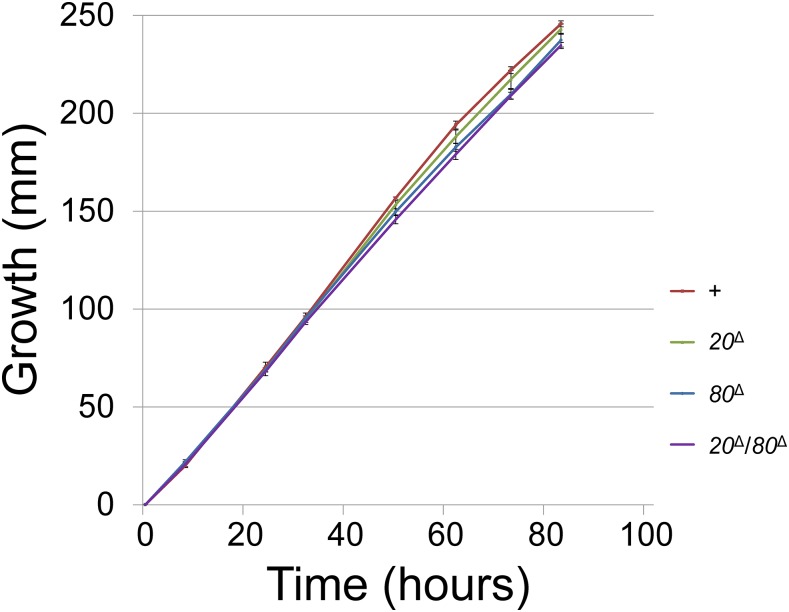
CBC is not essential for the vegetative stage. Mutations in *cbp20* and/or *cbp80* do not appear to have a substantial effect on the linear growth of the fungus. Strains used in this assay (performed in triplicate) include P3-08, P20-38, P20-47, and P20-68.

None of the known components of MSUD are essential for cell viability (Samarajeewa *et al.* 2014), and CBP20 and CBP80 are no exception (Figure 2). On the other hand, the first few reported MSUD proteins are all necessary for the sexual cycle. For example, the absence of either *qip* or *dcl-1* results in the production of perithecia that contain no asci, suggesting that they are important for early sexual development (Alexander *et al.* 2008; Xiao *et al.* 2010). A less severe phenotype can be seen in crosses lacking *sad-1*, *sad-2*, or *sad-3*, which are able to form asci that abort before ascospore production (Shiu *et al.* 2001, 2006; Hammond *et al.* 2011a). Hence, for a long period of time, it was thought that MSUD is required for sexual development. However, the discovery of *sad-4*, *sad-5*, and *sad-6* revealed this to be untrue, since crosses lacking any one of these genes are able to produce a decent number of mature ascospores (Hammond *et al.* 2013b; Samarajeewa *et al.* 2014).

**Figure 3:**
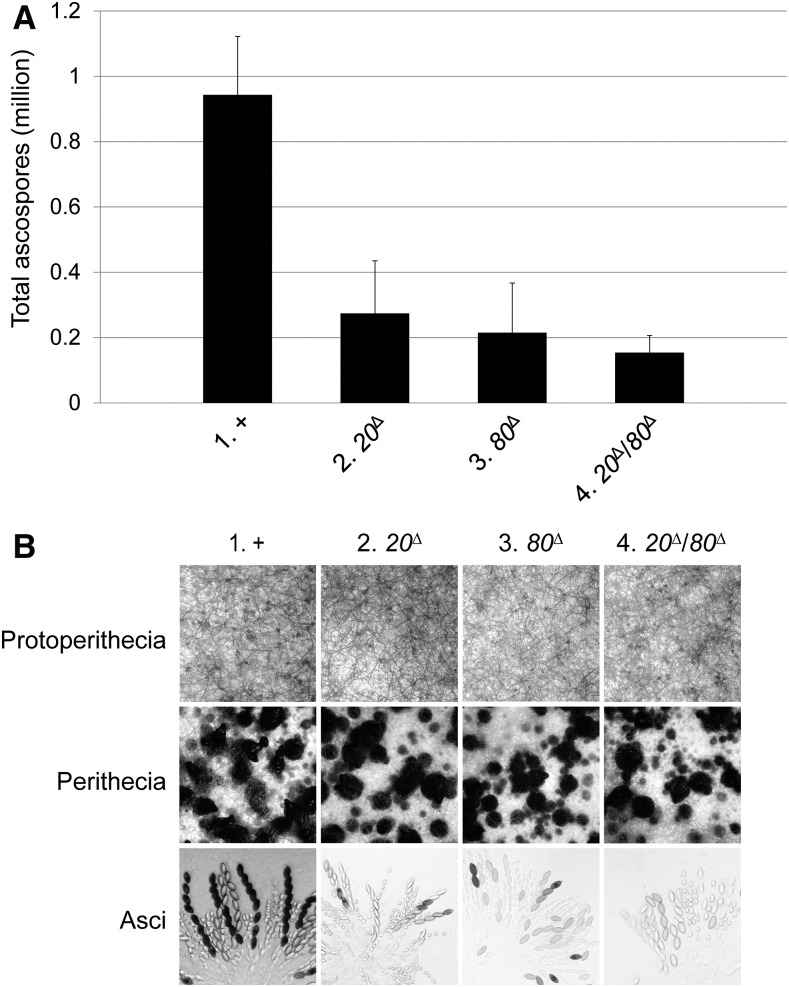
CBC-deficient crosses exhibit impaired sexual development. (A) The loss of CBP20 and/or CBP80 is associated with a considerable reduction in ascospore production. (B) Although protoperithecial development appears proficient in a given single or double *cbp* mutant, there only seem to be roughly half the normal number of mature perithecia in the corresponding homozygous cross. Dissection of mutant perithecia showed that they contain frequent ascus abortions. Deletion of *sad-5*, in comparison, does not significantly affect ascospore production (Hammond *et al.* 2013b). 1, F2-01 × P3-08. 2, F8-02 × P20-47. 3, F6-19 × P20-39. 4, F8-04 × P20-68.

To determine if CBC plays a role in sexual development, ascospore production was examined in a series of crosses. Although *cbp20* and *cbp80* are not absolutely required for sexual reproduction, their absence correlates with an appreciable decrease in the progeny count (Figure 3A). In fact, homozygous crosses of double knockouts (*cbp20*^∆^*/80*^∆^ × *cbp20*^∆^*/80*^∆^) yield only one-sixth of the normal number of ascospores, while those of a single knockout are somewhat more fertile. This CBC-dependent sporulation defect is reminiscent of that observed in yeast (Qiu *et al.* 2012). CBC-deficient crosses appear to have fewer mature perithecia and asci (Figure 3B).

### Localization of CBC components

**Figure 4:**
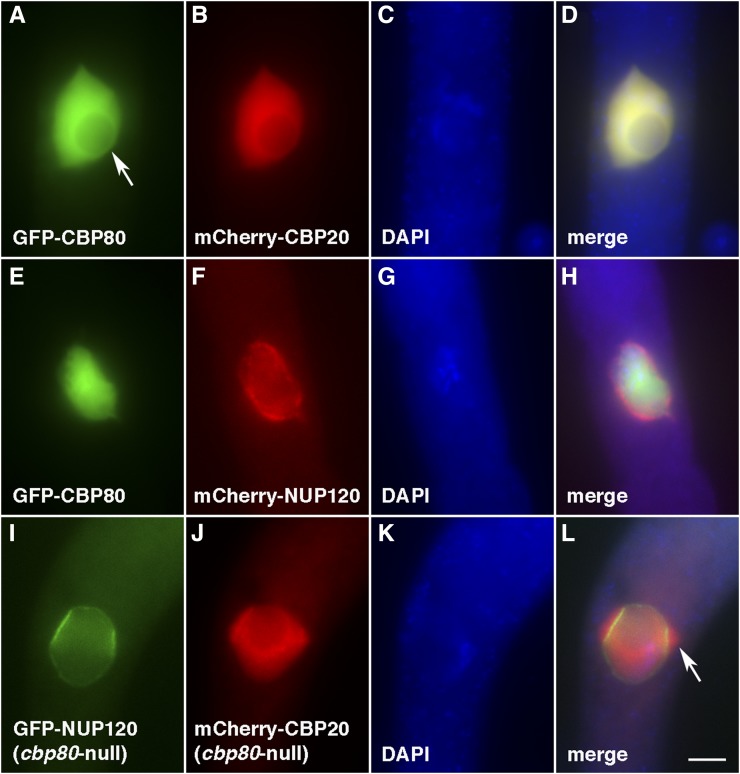
CBC localizes predominantly in the nucleus. (A–D) CBP20 and CBP80 colocalize in the nucleus, excluding the nucleolus (arrow). P20-44 × P20-45. (E–H) CBP80 does not appear to linger outside the nuclear envelope (labeled by nucleoporin NUP120). P20-34 × P20-35. (I–L) CBP20 accumulates outside the nuclear envelope in the absence of CBP80 (arrow), suggesting that its robust nuclear entry (and/or reentry) requires the latter. P23-28 × P23-29. The chromatin was stained with DAPI. Bar, 5 µm.

The first few described MSUD proteins localize in the perinuclear region, where they form a complex (MSC) to process RNAs exiting the nucleus (Shiu *et al.* 2006; Alexander *et al.* 2008; Xiao *et al.* 2010; Hammond *et al.* 2011a, b; Decker *et al.* 2015). The discovery of SAD-5 and SAD-6 demonstrated that some parts of the MSUD machinery are located inside the nucleus (Hammond *et al.* 2013b; Samarajeewa *et al.* 2014). Given CBC’s documented role in various nuclear processes in other organisms, it is conceivable that *Neurospora* CBP20 and CBP80 are also found mainly in the nucleus. To test this hypothesis, CBP20 and CBP80 were tagged with mCherry and green fluorescent protein (GFP), respectively. As expected, microscopic examination revealed that they localize predominantly in the nucleus (Figure 4, A–H). This result mirrors observations seen in other organisms, such as *Saccharomyces cerevisiae*, *Chironomus tentans*, *Homo sapiens*, and *Arabidopsis thaliana* (Görlich *et al.* 1996; Visa *et al.* 1996; Izaurralde *et al.* 1994; Kierzkowski *et al.* 2009). Not surprisingly, CBP20 colocalizes with CBP80, confirming that the two proteins occupy the same subcellular compartment (Figure 4D).

### CBP20 and CBP80 interact with each other and not with SAD-5

**Figure 5:**
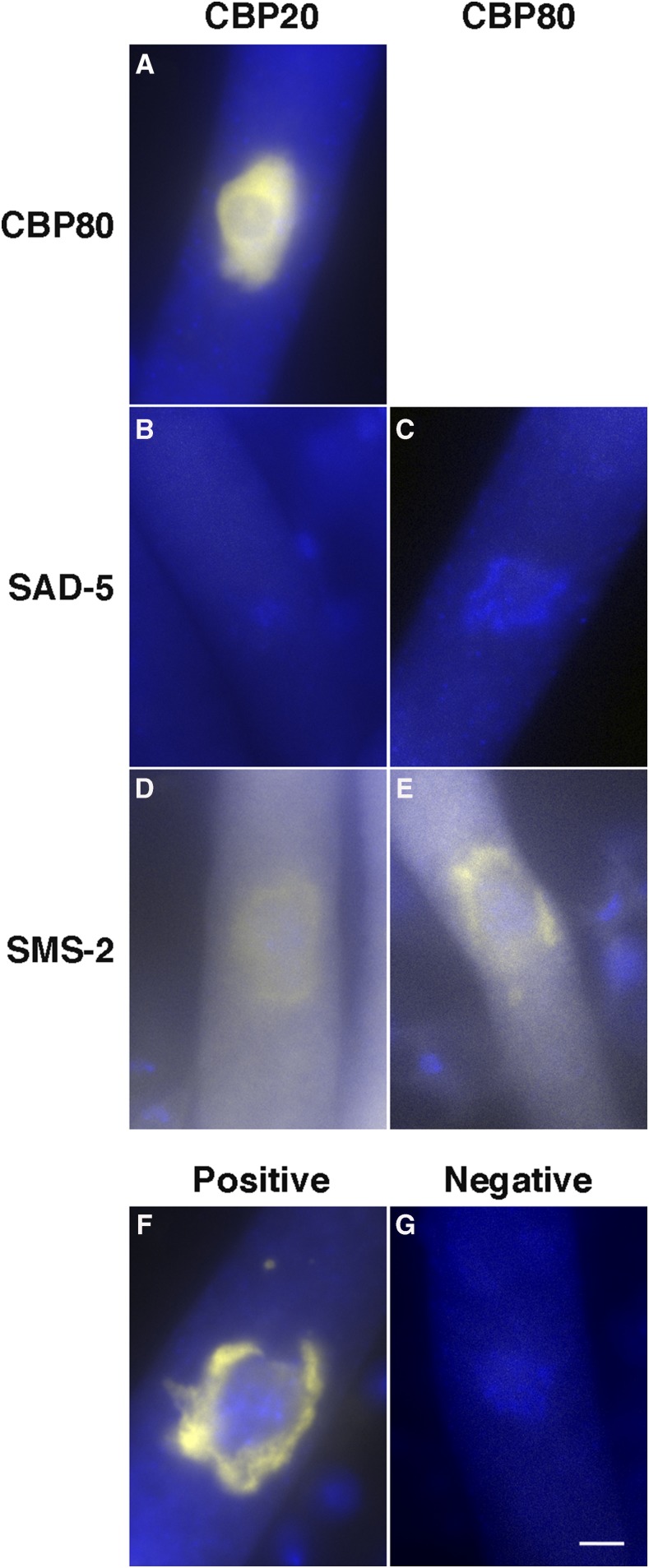
Interactions among CBP20, CBP80, and SMS-2. Similar to their eukaryotic counterparts, *Neurospora* CBP20 and CBP80 form a complex (A). The two cap-binding proteins interact with the SMS-2 Argonaute (D and E) and not SAD-5 (B and C). In a BiFC assay, each protein is tagged with either the N- or C-terminal fragment of YFP. A positive interaction (F) reconstitutes the yellow fluorophore, while a negative interaction (G) does not. Micrographs illustrate prophase asci expressing (A) *yfpn-cbp20* and *yfpc-cbp80* (P21-10 × P21-11), (B) *yfpn-cbp20* and *yfpc-sad-5* (P21-52 × P23-26), (C) *yfpn-cbp80* and *yfpc-sad-5* (P21-53 × P21-54), (D) *yfpn-cbp20* and *yfpc-sms-2* (P15-36 × P25-55), (E) *yfpn-sms-2* and *yfpc-cbp80* (P25-56 × P25-57), (F) *yfpn-sms-2* and *sad-1-yfpc* (P18-19 × P18-21), and (G) *yfpn* and *yfpc* (P6-59 × P8-25). The chromatin was stained with DAPI. Bar, 5 µm.

Since most eukaryotic CBP20 and CBP80 proteins are known to form a heterodimer, we set out to investigate whether the two *Neurospora* homologs have physical association during sexual development. Expectedly, we have shown that CBP20 and CBP80 have close interaction using BiFC (Figure 5A).

SAD-5 is another MSUD protein known to localize predominantly in the nucleus, although its precise function has yet to be established (Hammond *et al.* 2013b). We asked if SAD-5 is directly linked to CBC in the nuclear part of the MSUD mechanism. As shown in Figure 5 (B and C), SAD-5 does not interact with either CBP20 or CBP80, suggesting that it may be spatially separated from the CBC-related part of the silencing pathway.

### CBP20 requires CBP80 for efficient nuclear localization

Given the fact that CBP80 mediates the active nuclear import of CBP20 in animals (Izaurralde *et al.* 1995), it is possible for *Neurospora* to show a similar pattern. Our result indicates that CBP20 can be found in both nuclear and perinuclear regions when CBP80 is absent in a cross (Figure 4, I–L). This suggests that although CBP20 can independently enter the nucleus, its robust nuclear entry (and/or reentry) requires its heterodimeric partner.

### CBC interacts with a component of MSC

Since CBC travels in and out of the nucleus (Gonatopoulos-Pournatzis and Cowling 2014), we asked if it interacts with the perinuclear MSC. In eukaryotes, Argonaute is known as the core effector of the RNA-induced silencing complex, which uses small RNAs to target complementary transcripts. In a BiFC assay, we have shown that both CBP20 and CBP80 interact with the SMS-2 Argonaute, a component of MSC (Figure 5, D and E). This suggests that CBC mediates silencing through its interaction with MSC.

## Discussion

Like repeat-induced point mutation (RIP) (Cambareri *et al.* 1989) and quelling (Romano and Macino 1992), MSUD plays an important role in *Neurospora*’s genome surveillance (Aramayo and Selker 2013; Billmyre *et al.* 2013; Nicolás and Ruiz-Vázquez 2013). For example, it can target transposons and help restrict their transposition and expansion (Wang *et al.* 2015). This study links MSUD to CBC, a hub that connects various RNA pathways (Gonatopoulos-Pournatzis and Cowling 2014).

As expected, CBP20 and CBP80 directly interact to form CBC in *Neurospora*. This heterodimeric complex is predominantly nuclear. In eukaryotes, CBC is known to travel in and out of the nucleus to facilitate RNA export. As seen in animals but not in plants (Kierzkowski *et al.* 2009), robust nuclear import of CBP20 depends on CBP80 in *Neurospora*.

Similar to other reported MSUD components, CBC is not essential for somatic growth. However, it is one of the few MSUD players (along with quelling factors DCL-1 and QIP) that have a clear role during the vegetative cycle (*i.e.*, NMD). CBC is important for sexual development, as its absence is associated with a drastic reduction in ascospore production. Thus far, we have observed a range of sexual phenotypes in various silencing mutants (from complete ascus abortion to normal), and the results here further expand the complexity of how MSUD proteins may affect sexual development.

Our study shows that CBC has physical interaction with a component of MSC, thereby connecting the two formerly unrelated RNA-binding complexes. CBC is the first well-characterized RNA hub to be linked to MSUD, and it will be interesting to see if others follow suit. Although CBC and SAD-5 (a poorly defined MSUD protein) are both predominantly nuclear, they do not have direct interaction, leaving the role of SAD-5 still unclear.

Our current model holds that MSUD begins in the nucleus, where aRNAs are made from any gene lacking a pairing partner. These aRNAs are then exported to the perinuclear region, where they are converted into siRNAs that are capable of guiding MSC to target complementary mRNAs. An mRNA is normally bound at the 5′ cap by CBC, which aids its nuclear export. Since MSC resides just outside of the nuclear envelope and at least one of its components (SMS-2) interacts with CBC, one possibility is that MSC may efficiently detect exiting mRNAs by interacting with CBC. The absence of CBC may make it harder for MSC to recognize and capture target mRNAs, allowing some to reach the translational machinery and thus leading to a lower silencing efficiency. Alternatively, CBC may have a stimulatory effect on the silencing pathway directly or indirectly (Sabin *et al.* 2009). Future studies of CBC and related proteins should allow us to better define their connections to MSUD.
